# Antibodies to a Citrullinated *Porphyromonas gingivalis* Epitope Are Increased in Early Rheumatoid Arthritis, and Can Be Produced by Gingival Tissue B Cells: Implications for a Bacterial Origin in RA Etiology

**DOI:** 10.3389/fimmu.2022.804822

**Published:** 2022-04-20

**Authors:** Natalia Sherina, Charlotte de Vries, Nastya Kharlamova, Natalie Sippl, Xia Jiang, Boel Brynedal, Elin Kindstedt, Monika Hansson, Linda Mathsson-Alm, Lena Israelsson, Ragnhild Stålesen, Saedis Saevarsdottir, Rikard Holmdahl, Aase Hensvold, Gunnar Johannsen, Kaja Eriksson, Federica Sallusto, Anca I. Catrina, Johan Rönnelid, Caroline Grönwall, Tülay Yucel-Lindberg, Lars Alfredsson, Lars Klareskog, Luca Piccoli, Vivianne Malmström, Khaled Amara, Karin Lundberg

**Affiliations:** ^1^ Division of Rheumatology, Department of Medicine Solna, Center for Molecular Medicine, Karolinska Institutet, Karolinska University Hospital, Stockholm, Sweden; ^2^ Department of Clinical Neuroscience, Karolinska Institutet, Center for Molecular Medicine, Karolinska University Hospital, Stockholm, Sweden; ^3^ Institute of Environmental Medicine, Karolinska Institutet, Stockholm, Sweden; ^4^ Wallenberg Centre for Molecular Medicine, Umeå University, Umeå, Sweden; ^5^ Thermo Fisher Scientific, ImmunoDiagnositic Division, Uppsala, Sweden; ^6^ Department of Immunology, Genetics and Pathology, Uppsala University, Uppsala, Sweden; ^7^ Division of Clinical Epidemiology, Department of Medicine Solna, Karolinska Institutet, Stockholm, Sweden; ^8^ Faculty of Medicine, School of Health Sciences, University of Iceland, Reykjavik, Iceland; ^9^ Section for Medical Inflammation Research, Department of Medical Biochemistry and Biophysics, Karolinska Institutet, Stockholm, Sweden; ^10^ Center for Rheumatology, Academic Specialist Center, Stockholm Health Region, Stockholm, Sweden; ^11^ Division of Periodontology, Department of Dental Medicine, Karolinska Institutet, Stockholm, Sweden; ^12^ Danakliniken Specialisttandvård, Praktikertjänst AB, Danderyd, Sweden; ^13^ Division of Orthodontics and Pediatric Dentistry, Department of Dental Medicine, Karolinska Institutet, Stockholm, Sweden; ^14^ Institute for Research in Biomedicine, Universita dell a Svizzera Italiana, Bellinzona, Switzerland; ^15^ Institute of Microbiology, ETH Zurich, Zurich, Switzerland; ^16^ Centre of Occupational and Environmental Medicine, Region Stockholm, Stockholm, Sweden

**Keywords:** rheumatoid arthritis (RA), anti-citrullinated protein antibodies (ACPA), *Porphyromonas gingivalis (Pg)*, periodontitis (PD), monoclonal antibodies (mAbs), B cells

## Abstract

Based on the epidemiological link between periodontitis and rheumatoid arthritis (RA), and the unique feature of the periodontal bacterium *Porphyromonas gingivalis* to citrullinate proteins, it has been suggested that production of anti-citrullinated protein antibodies (ACPA), which are present in a majority of RA patients, may be triggered in the gum mucosa. To address this hypothesis, we investigated the antibody response to a citrullinated *P. gingivalis* peptide in relation to the autoimmune ACPA response in early RA, and examined citrulline-reactivity in monoclonal antibodies derived from human gingival B cells. Antibodies to a citrullinated peptide derived from *P. gingivalis* (denoted CPP3) and human citrullinated peptides were analyzed by multiplex array in 2,807 RA patients and 372 controls; associations with RA risk factors and clinical features were examined. B cells from inflamed gingival tissue were single-cell sorted, and immunoglobulin (Ig) genes were amplified, sequenced, cloned and expressed (n=63) as recombinant monoclonal antibodies, and assayed for citrulline-reactivities by enzyme-linked immunosorbent assay. Additionally, affinity-purified polyclonal anti-cyclic-citrullinated peptide (CCP2) IgG, and monoclonal antibodies derived from RA blood and synovial fluid B cells (n=175), were screened for CPP3-reactivity. Elevated anti-CPP3 antibody levels were detected in RA (11%), mainly CCP2+ RA, compared to controls (2%), p<0.0001, with a significant association to *HLA-DRB1* shared epitope alleles, smoking and baseline pain, but with low correlation to autoimmune ACPA fine-specificities. Monoclonal antibodies derived from gingival B cells showed cross-reactivity between *P. gingivalis* CPP3 and human citrullinated peptides, and a CPP3+/CCP2+ clone, derived from an RA blood memory B cell, was identified. Our data support the possibility that immunity to *P. gingivalis* derived citrullinated antigens, triggered in the inflamed gum mucosa, may contribute to the presence of ACPA in RA patients, through mechanisms of molecular mimicry.

## Introduction

Seropositive rheumatoid arthritis (RA) is characterized by presence of ACPA, autoantibodies targeting proteins citrullinated by peptidyl arginine deiminase (PAD) enzymes (1). ACPA associate with RA risk factors *HLA-DRB1* shared epitope (SE) and smoking (2), and with a more severe disease course (3). Data from experimental models suggest ACPA may directly contribute to pain and bone loss (4).

Since ACPA can be detected years before clinical symptoms (5), it has been suggested that break of tolerance to citrullinated proteins may occur at extra-articular (mucosal) sites. While many studies have focused on the lungs (2, 4, 6), the gum mucosa has raised increasing interest in this context. There is an epidemiological link between periodontitis (PD), gingival inflammation causing tooth loss as a result of degraded jawbone, and RA (7). Jawbone loss has also been found increased in ACPA+ individuals before RA symptom onset (8), and higher prevalence of PD was reported for ACPA+ at-risk of RA individuals *versus* population controls (9, 10), implicating that PD may precede RA onset.

Notably, citrullinated proteins are present in PD gingival tissue (GT), and increased PAD activity has been detected in PD gingival crevicular fluid (11, 12). Moreover, *Porphyromonas gingivalis* (*Pg*), a key pathogen driving PD (13), is the only pathogen known to express a PAD enzyme (*P*.PAD) (14), capable of citrullinating bacterial as well as host proteins, including the RA candidate autoantigens fibrinogen, α-enolase, vimentin and histones (15–18). A number of reports also suggest *P.*PAD can autocitrullinate (14, 15, 17–19). With citrullinated proteins exposed in the bacteria-rich inflamed gingiva, we hypothesize that this milieu may facilitate break of tolerance and production of ACPA (20), possibly by mechanisms of molecular mimicry. Notably, we have previously shown that circulating ACPA targeting human citrullinated α-enolase cross-react with the *Pg* counterpart (21).

Increased antibody responses to *Pg* antigens, including citrullinated *P.*PAD, have been shown in RA, up to 10 years before diagnosis, and in individuals at-risk of RA, compared to population controls (15, 17, 22–24). However, it has not been reported how the antibody response to citrullinated *P.*PAD relates to the autoimmune ACPA response, RA risk factors and clinical features, or whether such antibodies are produced locally in the inflamed periodontium of PD patients. Hence, in the present study, we have investigated the antibody response to citrullinated *Pg* PAD in a large and well-characterized early RA cohort, by screening for reactivity against a citrullinated *P.*PAD peptide denoted CPP3, previously identified as an immunodominant epitope in RA (15, 23). Additionally, for the first time we have generated single-cell derived monoclonal antibodies (mAbs) from gingival tissue B cells of PD patients, and analyzed their reactivity to *Pg* and human citrullinated antigens.

## Materials and Methods

### Study Population

Newly diagnosed, disease modifying anti-rheumatic drug (DMARD)-naïve, RA patients and population controls from the Epidemiological Investigation of RA (EIRA) study (25) were included. Data on *HLA-DRB1* SE and *PTPN22* (2,807 RA; 1,936 controls), smoking (2,807 RA; 4,864 controls), anti-CCP2 IgG (2,859 RA), and 5-year clinical data (1,600 RA), captured through linkage with the Swedish Rheumatology Quality Register, were generated previously and retrieved from the EIRA database (2, 26, 27). Periodontal status was not available for EIRA cases and controls.

Biopsies were obtained from inflamed gingival tissue (GT) during surgical treatment of periodontitis (n=3 ACPA+ RA/PD patients; n=4 non-RA/PD patients) at the Department of Dental Medicine, Karolinska Institutet, and specialist clinic Danakliniken Danderyd, Sweden.

All RA patients fulfilled the ACR 1987 and/or ACR/EULAR 2010 RA classification criteria. Samples were collected in compliance with the Declaration of Helsinki, with informed consent and ethical approval (Regional Ethics Review Board Stockholm).

### Isolation of Gingival Tissue B Cells and Generation of Monoclonal Antibodies

Gingival tissue biopsies were either collected in ice-cold PBS, 2% human sera (HS), and processed directly (n=4: GT01, GT03, GT09 and GT10), or snap-frozen in liquid nitrogen and stored at -80°C (12-184 days) before processed (n=4: GT05-GT08). According to a modified protocol (28), the gingival tissue biopsies were rinsed in PBS, 2% HS, digested mechanically on ice (in 200µl PBS, 2% HS) and treated with 0.5mg Liberase™ Dispase-High (RocheDiagnostics GmbH, Mannheim, Germany) for 1h at 37°C, 200rpm. Dissociated GT cells were then labelled for flow cytometry analysis and single-cell sorting. See **Supplementary Methods** for detailed staining protocols.

Single viable CD3-/CD14-/CD19+ B cells were sorted into 96-well PCR plates containing 5μl 0.5×PBS, 10mM DTT, 8U RNAsin inhibitor/well (Promega, Madison, WI, USA); variable region immunoglobulin (Ig) cDNA was synthesized by reverse transcription and amplified using multiplex PCR, as previously described (29). Immunoglobulin heavy (IgH) and light (IgL) chains were sequenced (Eurofins Genomics) and annotated using IgBLAST, and IMGT/V-QUEST; variable region N-linked glycosylation sites were identified in translated sequences of V-(D)-J regions using the NetNGlyc1.0 server (www.cbs.dtu.dk/services/NetNGlyc/).

Recombinant human monoclonal antibodies (mAbs) were generated from one ACPA+RA/PD patient (GT01; fresh biopsy) and one non-RA/PD patient (GT06; frozen biopsy) as previously described (29–31). In brief, Ig gene-specific PCR was run to introduce restriction sites for expression vector cloning. Purified digested PCR products were ligated into expression vectors containing human Igγ1, Igλ or Igκ constant regions, and transformed into DH5α bacteria; purified IgH and IgL plasmids were sequenced to confirm identity with the original PCR products, and mAbs were produced by transient co-transfection in high-density suspension cultures of Expi293 cells using the polyethyleneimine (PEI)-precipitation method. Supernatants were collected after 5 days of culture, and mAbs purified on protein G-Sepharose^®^ 4 Fast flow beads, eluted with 0.1M glycine buffer into 1M Tris-HCl, pH 8.0, following buffer exchange to PBS. IgG1 concentrations were determined by IgG ELISA, as previously described (29). Monoclonal antibodies derived from RA synovial fluid (n=139) and peripheral blood (n=36) plasma or memory B cells from 11 ACPA+ RA patients, including 13 CCP2+ clones, were produced previously, following the same protocol (29–33).

### Citrulline-Reactivity by ELISA and Multiplex Microarray

Sera from 2,859 RA patients and 372 controls were screened on a custom-made multiplex microarray (ThermoFisher Scientific, ImmunoDiagnostics Division, Uppsala, Sweden) for IgG reactivity against citrullinated peptides derived from *Pg* PAD (CPP3) and human proteins (**Supplementary Table 1**), as previously described (26, 34). Two different CPP3-cutoff values, corresponding to the 98^th^ and 80^th^ percentile reactivity among controls, were used.

Monoclonal antibodies (5µg/ml) were assayed for CCP2-reactivity using the Immunoscan CCPlus^®^ assay (Euro-Diagnostica, Malmoü, Sweden), in accordance with the kit instructions, and for reactivity against CPP3 and human protein-derived citrullinated peptides (**Supplemental Table 1**) using in-house ELISAs as previously described (15, 23, 30, 35). See **Supplementary Methods** for a detailed protocol. Polyreactivity/unspecific binding was analyzed using uncoated wells blocked with 2% BSA, and in LPS-, insulin-, and dsDNA ELISAs, following established protocols (29). In addition, polyreactivity was evaluated in an ELISA based on the soluble membrane protein fraction of Hek293 cells (fractionated using ProteoExtract subcellular proteome extraction kit, Calbiochem, Darmstadt, Germany), as previously described (36). See **Supplementary Methods** for a detailed protocol.

Affinity-purified anti-CCP2 IgG, and corresponding anti-CCP2 IgG-depleted IgG pools (35), were also analyzed in the CPP3 ELISA.

### Statistical Methods

Mann-Whitney U test was used to assess anti-CPP3 IgG levels as well as clinical variables, with adjustment for age, sex and smoking (in multivariant analyses). ACPA co-occurrence was calculated using pairwise Pearson correlation (using Rv.3.3.3), among RA patients expressing > one ACPA fine-specificity. Associations between RA risk factors and anti-CPP3 IgG was calculated in SAS (version 9.4) using unconditional logistic regression and presented as odds ratios (OR) with 95% confidence intervals (CI); adjusted for age, sex and residential area. Statistical differences for Ig gene characteristics were not evaluated due to the limited number of patients (n=2).

## Results

### CPP3-Reactivity in Relation to CCP2 and the Autoimmune ACPA Response in RA

**Figure 1:**
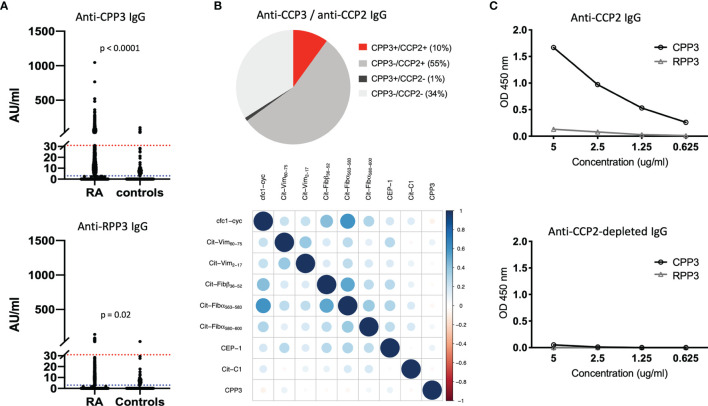
The anti-CPP3 antibody response in relation to the autoimmune ACPA response. **(A)** Anti-CPP3 (top panel) and anti-RPP3 (bottom panel) IgG levels in EIRA RA patients (n=2,859) and controls (n=372), measured by multiplex microarray; 98^th^ percentile cut-off (red line), and 80^th^ percentile cut-off (blue line) are shown; p-values indicate differences in CPP3/RPP3 IgG levels between groups. **(B)** Pie chart illustrating the frequency (%) of CPP3+ RA patients (98^th^ percentile cut-off) in relation to CCP2 status (n=2,859) (top panel), and co-occurrence of anti-CPP3 IgG (98^th^ percentile cut-off) in relation to eight autoimmune ACPA fine-specificities (measured by multiplex microarray) in EIRA RA patients (n=2,859) (bottom panel); correlation between antibody expression vectors (corrplot package v. 0.77) was used to illustrate co-occurrence, colours signify degree of correlation according to Pearson correlation coefficient (full scale shown on the right). **(C)** CPP3/RPP3 reactivity in a pool of affinity-purified anti-CCP2 IgG (top panel) and corresponding anti-CCP2 IgG-depleted IgG pool (bottom panel), at different IgG concentrations. AU, arbitrary units; CPP3, citrullinated *P.*PAD peptide; RPP3, arginine-containing version of CPP3; RA, rheumatoid arthritis; CCP2, cyclic citrullinated peptide(s); OD, optical density; cfc1-cyc, cyclic citrullinated filaggrin peptide 1; Cit-Vim, citrullinated vimentin; Cit-Fib, citrullinated fibrinogen; CEP-1, citrullinated α-enolase peptide 1; Cit-C1, citrullinated triple helical C1 epitope on collagen type II.

We first screened 2,859 early RA serum samples on a multiplex array containing *Pg* CPP3 and eight citrullinated peptides derived from human proteins previously identified as RA candidate autoantigens (**Supplementary Table 1**). With a cut-off set at the 98^th^ percentile among controls, 11% of RA patients were anti-CPP3 IgG positive, with no significant reactivity to the corresponding non-modified counterpart RPP3 (**Figure 1A**).

Since CPP3/RPP3 are oral bacteria-derived antigens, we hypothesize that some CPP3/RPP3 reactivity seen in controls may reflect a true bacterial immune response rather than background reactivity. Hence, we also analyzed the dataset using a lower cut-off (80^th^ percentile), allowing 20% CPP3+ controls. With this lower cut-off, anti-CPP3 IgG was detected in 42% of RA patients, while anti-RPP3 IgG was detected in 11% of RA and 15% of controls. Both anti-CPP3 and anti-RPP3 IgG levels were significantly higher in RA patients than in controls.

Similar to the autoimmune ACPA fine-specificities (26), a majority of CPP3+ RA patients (91%) were confined to the CCP2+ subset, but showed weak correlation to other ACPA-reactivities (Pearson correlation coefficients between -0.074 and 0.098) (**Figure 1B**).

With access to affinity-purified polyclonal anti-CCP2 IgG (35), we could also show binding to CPP3 but not RPP3, demonstrating cross-reactivity between *Pg* CPP3 and CCP2, i.e. the antigenic peptide(s) used in the gold standard clinical ACPA test (**Figure 1C**).

### Anti-CPP3 Antibodies in Relation to RA Risk Factors and Clinical Features

To further characterize the CPP3+ RA subset, we analyzed RA risk factors. HLA-*DRB1* SE showed a stronger association with CPP3+ (OR=6.07) compared to CPP3- RA (OR=2.55), p<0.0001 (**Table 1**). However, the significance for the association was lost when analyzing the CCP2+ subset only. *PTPN22* polymorphism associated with both CPP3+ (OR=1.85) and CPP3- RA (OR=1.60), with no difference between subsets (p=0.5). Smoking on the other hand, showed a stronger association with CPP3+ compared to CPP3- RA, even within the CCP2+ subset (OR=2.88 for CPP3+ RA *vs*. OR=1.75 for CPP3- RA, p=0.0012).

**Table 1:** Associations between anti-CPP3 IgG and RA risk factors in EIRA.

Subgroup	No SE (%)	Any SE (%)	OR (95% CI)*	P-values
Controls	959 (49.87)	964 (50.13)	1.0 ref.	
CPP3-	563 (28.09)	1441 (71.91)	**2.55** (2.22-2.93)	**<0.0001**
CPP3+	36 (14.57)	211 (85.43)	**6.07** (4.19-8.78)	
CCP2-/CPP3-	374 (47.22)	418 (52.78)	1.11 (0.94-1.32)	
CCP2-/CPP3+	8 (32.00)	17 (68.00)	2.07 (0.87-4.88)	
CCP2+/CPP3-	189 (15.59)	1023 (84.41)	**5.52** (4.58-6.64)	0.32
CCP2+/CPP3+	28 (12.61)	194 (87.39)	**7.27** (4.82-10.97)	
	**No *PTPN22*** (%)	**Any *PTPN22*** (%)		
Controls	1533 (79.18)	403 (20.82)	1.0 ref.	
CPP3-	1574 (70.36)	663 (29.64)	**1.60** (1.38-1.85)	0.5
CPP3+	179 (68.32)	83 (31.68)	**1.85** (1.38-2.47)	
CCP2-/CPP3-	634 (73.64)	227 (26.36)	**1.38** (1.14-1.67)	
CCP2-/CPP3+	13 (56.52)	10 (43.48)	**3.49** (1.47-8.24)	
CCP2+/CPP3-	940 (68.31)	436 (31.69)	**1.76** (1.50-2.07)	0.72
CCP2+/CPP3+	166 (69.46)	73 (30.54)	**1.74** (1.28-2.35)	
	**Never smokers** (%)	**Ever smokers** (%)		
Controls	2114 (43.46)	2750 (56.54)	1.0 ref.	
CPP3-	812 (32.32)	1700 (67.68)	**1.50** (1.35-1.67)	**<0.0001**
CPP3+	63 (21.36)	232 (78.64)	**2.73** (2.04-3.66)	
CCP2-/CPP3-	360 (37.31)	605 (62.69)	**1.18** (1.02-1.37)	
CCP2-/CPP3+	8 (29.63)	19 (70.37)	1.76 (0.75-4.11)	
CCP2+/CPP3-	452 (29.22)	1095 (70.78)	**1.75** (1.54-1.99)	**0.0012**
CCP2+/CPP3+	55 (20.52)	213 (79.48)	**2.88** (2.11-3.92)	

*Odds ratios (OR) were adjusted for age, sex and residential area. Significant values are shown in bold. P-values show differences between CPP3+ and CPP3- or CCP2+/CPP3+ and CCP2+/CPP3- RA (CPP3 IgG status based on the 98^th^ percentile cut-off). P-values were not calculated for CCP2-/CPP3- versus CCP2-/CPP3+ RA, due to the low number of CCP2-/CPP3+ patients. No/Any, No risk alleles/one or two risk alleles. Ever smokers include current and former smokers.

We also co-analyzed clinical features. Patient-reported baseline pain and global assessment were significantly higher in CPP3+ compared to CPP3- RA, but notably not in CCP2+ compared to CCP2- RA (**Table 2**). The difference for pain, but not patient global, remained significant after adjustment for age, sex and smoking. The same results were obtained when anti-CPP3 IgG was analyzed within the CCP2+ subset (**Supplementary Table 2**). Anti-CPP3 IgG also associated with significantly higher ESR at 12 and 48 months, but not with CRP, swollen-/tender joint counts, disease activity score in 28 joints (DAS28) or the health assessment questionnaire (HAQ). Most of these other clinical variables were significantly higher in CCP2+ *versus* CCP2- RA over time (**Supplementary Tables 3–6**).

**Table 2:** Baseline pain and global assessment, stratified for CPP3 or CCP2 status in EIRA.

Baseline Pain (median VAS)					
CPP3 + (N=173)	CPP3- (N=1380)	P-values	CCP2 + (N=990)	CCP2- (N=563)	P-values
60	50	**0.009**	51	51	0.619*
		0.020*			0.682*
		0.021**			0.677**
**Baseline global assessment** (median VAS)					
**CPP3+** (N=173)	**CPP3-** (N=1380)	**P-values**	**CCP2+** (N=990)	**CCP2-** (N=563)	**P-values**
56	51	**0.036**	51	51	0.957
		0.047*			0.915*
		0.053**			0.812**

Median values for patient-reported pain and global assessment (using visual analogue scale: 0-100) at baseline, in CPP3+ versus CPP3- RA (CPP3 IgG status based on the 98^th^ percentile cut-off), and in CCP2+ versus CCP2- RA. Significant p-values are shown in bold. *p-values adjusted for smoking status, **p-values adjusted for age, sex and smoking status (multivariate analyses). N, number of patients with clinical data available.

### Isolation of Gingival Tissue B Cells From PD Patients With and Without ACPA+ RA

**Figure 2:**
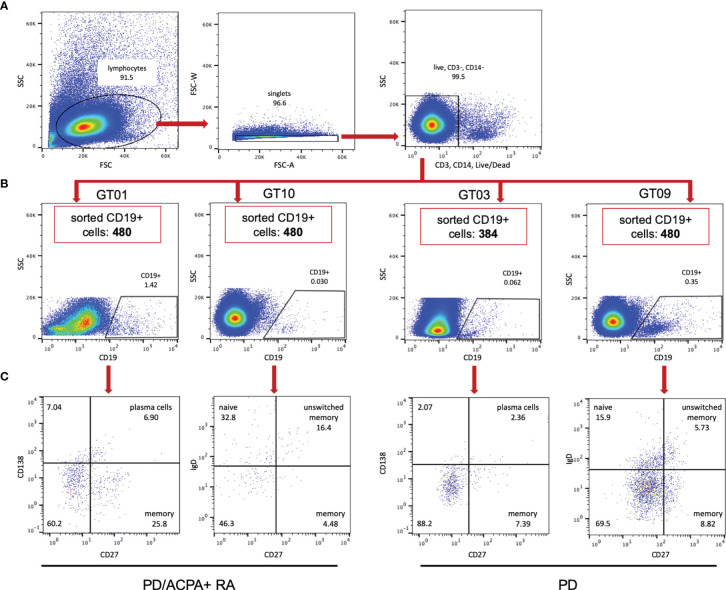
Flow cytometry sorting of CD19+ B cells from gingival tissue biopsies. **(A)** Representative general gating strategy for CD19+ B cells from fresh GT biopsies. **(B)** Flow cytometry plots showing the frequency of CD19+ B cells after exclusion of dead, CD3+ and CD14+ cells; GT01 and GT10 originate from PD patients with ACPA+ RA (PD/ACPA+ RA), GT03 and GT09 originate from PD patients without RA (PD). **(C)** Flow cytometry plots showing the frequency of memory B cells (CD19+/CD27+/CD138-) and plasma cells (CD19+/CD27+/CD138+) in GT01 and GT03, or memory B cells (CD19+/CD27+/IgD-), naïve B cells (CD19+/CD27-/IgD+) and unswitched memory B cells (CD19+/CD27+/IgD+) in GT10 and GT09. Red boxes show the number of CD19+ B cells sorted from each biopsy. FSC, forward scatter; SSC, side scatter; GT, gingival tissue; PD, periodontitis; ACPA, anti-citrullinated protein antibody; RA, rheumatoid arthritis.

We next set out to investigate presence of *Pg* CPP3-reactive B cells in the gum mucosa. Thus, we sorted single B cells from four fresh (**Figure 2**) and four frozen (**Supplementary Figure 1**) gingival tissue biopsies, of PD patients with (n=3) and without (n=4) ACPA+ RA, and retrieved between 108 and 576 live CD3-/CD14-/CD19+ cells from each biopsy. Cell yields from frozen samples were variable and consistently lower than from fresh samples, and the lymphocyte population was generally less clear, hence B-cell subsets were analyzed in fresh samples only.

Using two different B-cell panels, we compared memory and plasma cell frequencies in one ACPA+ RA/PD (GT01; fresh biopsy) *versus* one non-RA/PD (GT03; fresh biopsy) patient, and memory, naïve and unswitched memory B cells in another ACPA+ RA/PD (GT10; fresh biopsy) *versus* non-RA/PD (GT09; fresh biopsy) patient. With the first panel, memory B cells (CD19+/CD27+/CD138-) and plasma cells (CD19+/CD27+/CD138+) were detected in both the ACPA+ RA and non-RA sample. With the other panel, we detected memory B cells (CD19+/CD27+/IgD-), naïve (CD19+/CD27-/IgD+), and unswitched memory B cells (CD19+/CD27+/IgD+) in both the ACPA+ RA and non-RA sample.

### Gingival Tissue B-Cell Receptor (Ig) Repertoire

We subsequently analyzed GT B-cell receptor variable gene region sequences, and distribution of Ig subclasses, in one ACPA+ RA/PD patient (GT01; fresh biopsy) and one non-RA/PD patient (GT06; frozen biopsy), selected based on the successful recovery of heavy and light chain PCR product from the sorted B cells. Ninety-four matched variable heavy (VH) and light (VL) sequences were generated from GT01, and 54 from GT06. VH3 gene family representation was higher in the ACPA+ RA/PD patient, and by individual variable region genes, IGHV4– 31, IGKV1-33 and IGLV1-47 were overrepresented, while IGHV4-4, IGHV4-38, IGHV1-69 and IGLV1-51 were underrepresented (**Supplementary Figure 2**).

The complementary determining region 3 (CDR3) length and number of negatively and positively charged amino acids were similar, and the GT immunoglobulins displayed similarly high numbers of somatic hypermutations (SHM), with a median of 27.0 (VH) and 20.0 (VL) for the ACPA+ RA/PD patient, and 27.5 (VH) and 16.0 (VL) for the non-RA/PD patient; mutations were distributed within the variable region with enrichment of replacement (R) over silent (S) mutations in the CDRs. The frequency of N-glycosylation sites was higher in the ACPA+ RA/PD patient (24.5%) than in the non-RA/PD patient (14.7%). The distribution of Ig subclasses was dominated by IgG1 followed by IgA in the ACPA+ RA/PD patient, and IgG1 and IgG2 in the non-RA/PD patient, with lower numbers of IgG3 and IgG4 (**Supplementary Figure 3**).

### Expression of Gingival Tissue Monoclonal Antibodies and Detection of Citrulline-Reactivity

To assess presence of citrulline-reactive GT B cells, we randomly selected B-cell clones from the ACPA+ RA/PD patient (GT01; fresh biopsy) and recombinantly expressed 70 mAbs. We also expressed 16 GT mAbs from the non-RA/PD patient (GT06; frozen biopsy) after preselecting clones with a high level of SHM (>15) and/or N-glycosylation sites [i.e. previously described ACPA characteristics (30, 31, 33)]. Antibodies expressed at >100 μg/ml (52 from the ACPA+ RA/PD patient; 11 from the non-RA/PD patient) were analyzed.

**Figure 3:**
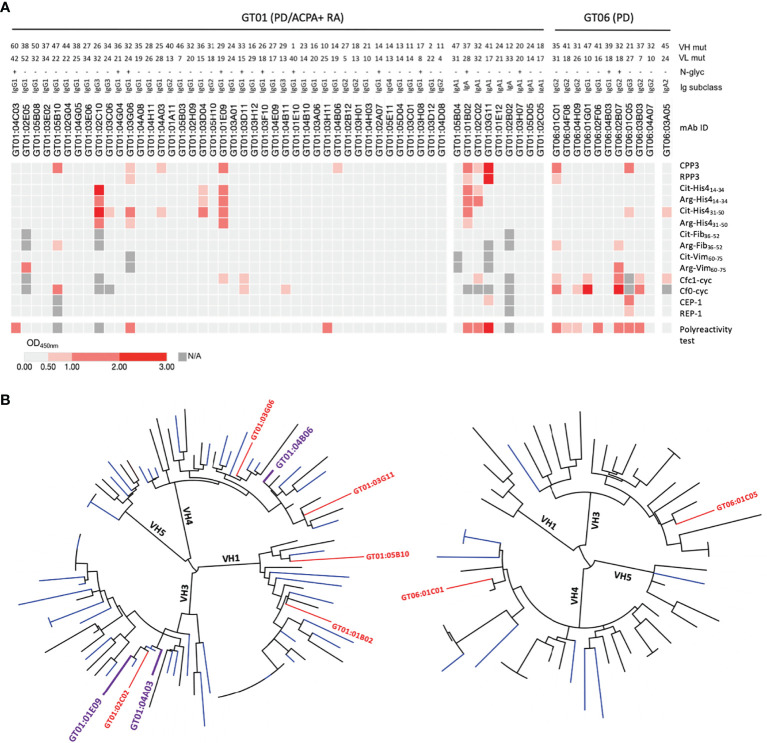
Recombinant gingival tissue mAbs exhibit citrulline-reactivity. **(A)** ELISA results shown as a heat map, for 52 mAbs generated from a fresh GT biopsy of a PD patient with ACPA+ RA (GT01) and 11 GT mAbs generated from a frozen GT biopsy of a PD patient without RA (GT06); colors indicate binding strength (based on OD values; scale shown at the bottom). Peptide antigens include: citrullinated peptides (and arginine-containing equivalents) from *Pg* (CPP3) and human proteins. The number of mutations (mut) in the variable heavy (VH) and variable light (VL) chains, presence/absence (+/-) of predicted N-linked glycosylation (N-glyc) motifs (N-X-S/T), and the original Ig subclasses are shown. **(B)** Phylogenetic trees of the VH chain sequences of GT01 (94 sequences) (left), and GT06 (54 sequences) (right), constructed using Phylogeny.fr with MUSCLE alignment and visualized by iTOL v4.4.2. CPP3+ clones are highlighted in plum (no polyreactivity detected) or in red (show polyreactivity). CPP3, citrullinated *P*.PAD peptide 3; RPP3, arginine-containing version of CPP3; Cit, citrulline; Arg, arginine; His4, histone-4; Fib, fibrinogen; Vim, vimentin; Cfc1-cyc, cyclic citrullinated filaggrin peptide 1; Cf0-cyc; arginine-containing version of Cfc1-cyc; CEP-1, citrullinated α-enolase peptide 1; REP, arginine-containing version of CEP-1; OD, optical density; N/A, not analysed. Polyreactivity test, ELISA based on the soluble membrane protein fraction of Hek293 cells.

Our mAb ELISA data show that 10 GT B cells were positive for the *Pg* CPP3 peptide derived from *P*.PAD (**Figure 3A**). Four CPP3+ mAbs also showed reactivity with the non-modified counterpart RPP3. Additionally, eight CPP3+ clones (and seven CPP3- clones) showed reactivity with citrullinated peptides derived from human proteins, mainly histone-4 and filaggrin, but for most of these peptide-reactivities, also the arginine counterparts were targeted. None of the mAbs were polyreactive/unspecific when analyzed by LPS-, insulin- and dsDNA ELISAs (data not shown). However, when tested in an ELISA based on soluble HEK293 cell membrane proteins, 6/53 clones from GT01 and 7/11 clones from GT06 were positive, suggesting an enrichment of unspecific autoreactivity in preselected clones from the non-RA/PD patient (GT06; frozen biopsy). Still, three CPP3+ clones from the ACPA+ RA/PD patient (GT01; fresh biopsy) had no polyreactivity or RPP3-reactivity. Two of these clones (GT01:04A03 and GT01:01E09) showed cross-reactivity with citrullinated filaggrin and/or histone-4 peptides. None of the clones were positive for CCP2 (data not shown). Phylogenetic trees of VH gene sequences showed that 9.6% of the ACPA+ RA/PD and 3.7% of the non-RA/PD GT B cells were clonally related (**Figure 3B**).

### Identification of a CPP3/CCP2 Cross-Reactive Memory B Cell in RA Peripheral Blood

**Figure 4:**
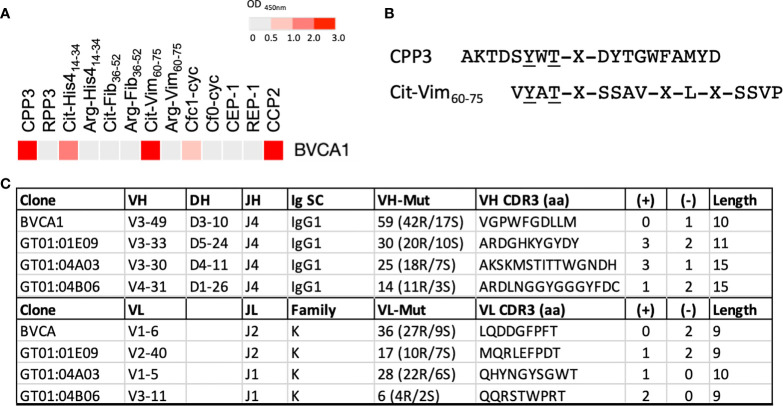
Binding pattern for BVCA1, CPP3/Cit-Vim_60-75_ peptide alignment, and Ig gene features of BVCA1 *versus* CPP3+ GT mAbs. **(A)** ELISA results for BVCA1 shown as a heat map; colors indicate binding strength (based on OD values; scale on top). **(B)** Peptide alignment of the citrullinated *P*.PAD peptide CPP3 and a citrullinated human vimentin peptide (Cit-Vim_60-75_); identical amino acids underlined, X=citrulline. **(C)** Immunoglobulin (Ig) heavy (H) and light (L) chain gene features of BVCA1 and three CPP3+ GT mAbs; Ig gene variable (V), diversity (D) and joining (J) segment usage, Ig subclass (SC), L chain family, complementary determining region (CDR3) features, and number of mutations (mut) are shown. CPP3, citrullinated *P*.PAD peptide 3; RPP3, arginine-containing version of CPP3; Cit, citrulline; Arg, arginine; His4, Histone-4; Fib, Fibrinogen; Vim, Vimentin; Cfc1-cyc, cyclic citrullinated filaggrin peptide 1; Cf0-cyc; arginine-containing version of Cfc1-cyc; CEP, citrullinated α-enolase peptide 1; REP, arginine-containing version of CEP-1; CCP2, cyclic citrullinated peptide(s); S, silent; R, replacement; aa, amino acids; (+)/(-), positively/negatively charged aa; Length, number of aa in the CDR3 region.

To investigate presence of CPP3+ B cells in the circulation and joints of RA patients, we next analyzed in-house generated mAbs derived from single peripheral blood (PB) (n=36) and synovial fluid (n=139) plasma and memory B cells of 11 ACPA+ RA patients, including 13 CCP2+ clones (30, 31, 33), (**Supplementary Table 7**). We identified one CCP2+ PB mAb (denoted BVCA1) with strong CPP3-reactivity and no unspecific polyreactivity. BVCA1, originally identified as a strong binder to a citrullinated vimentin peptide (Cit-Vim_60-75_), also showed multireactivity to citrullinated histone-4 and filaggrin peptides (**Figure 4A**). Thus, BVCA1 demonstrates cross-reactivity on the monoclonal level, between a bacterial epitope, human citrullinated peptides, and the CCP2 peptide(s). Peptide alignment of CPP3 and Cit-Vim_60-75_ revealed two identical amino acids adjacent to citrulline (**Figure 4B**). Comparison of SHM show more mutations and higher R/S ratio in the CPP3+/CCP2+ BVCA1 clone, compared to the CPP3+/CCP2- GT clones (**Figure 4C**).

## Discussion

Based on an epidemiological link between PD and ACPA+ RA, and the unique feature of the periodontal pathogen *Porphyromonas gingivalis* to generate citrullinated proteins, it has been hypothesised that break of tolerance to citrullinated proteins and production of ACPA may occur in the gum mucosa (7, 14, 20). With the present study, we show an antibody response against the citrullinated *Pg* PAD enzyme in a subset of early RA, and presence of citrulline-reactive gingival B cells, which display cross-reactivity between *Pg* and human peptide antigens, supporting the concept of a bacterial/periodontal origin for the ACPA response.

We have focused our investigations on the antibody response to citrullinated *P*.PAD, first described in RA by Quirke et al. (15). It has been suggested that citrullinated *P.*PAD could be generated *in vivo* by autocitrullination, as studies of recombinant full-length *P.*PAD show autocitrullination (14, 15, 37), similar to human PAD enzymes (38). However, during infection, *P.*PAD is secreted in vesicles in a truncated form (14, 39, 40) and Konig et al. could show that the truncated form was not autocitrullinated (37), questioning whether autocitrullination would occur *in vivo*. Still, autocitrullination of the truncated form was reported by Rodriguez et al. (19) and more recent studies show autocitrullinated *P.*PAD in clinical *Pg* isolates (17, 18). Citrullination of *P.*PAD could possibly also occur in the context of NETosis, which is extensive during PD, when secreted *P.*PAD is likely in close proximity to human PAD enzymes (41). Based on these different scenarios, we hypothesise that an antibody response towards citrullinated *P.*PAD could be triggered in the inflamed periodontium during *Pg* infection.

In the large EIRA cohort (2,807 RA patients and 372 controls), we found high serum levels of antibodies to the citrullinated *Pg* CPP3 peptide derived from *P*.PAD only in RA patients, but low levels of anti-CPP3 IgG were detected also in a subset of controls. Additionally, low levels of antibodies to the corresponding arginine-containing peptide RPP3 were observed in both RA patients and controls. Periodontal status in EIRA is not known, but we hypothesize that the CPP3+/RPP3+ subsets have (or have had) a *Pg* infection. In support of this hypothesis, we have previously shown an anti-CPP3 IgG response in rats after *Pg* infection (42).

High anti-CPP3 IgG levels were mainly recorded in the CCP2+ subset, but did not show a strong correlation with the autoimmune ACPA fine-specificities. Moreover, anti-CPP3 IgG associated with *HLA-DRB1* SE, but the association was likely dependent on the co-occurrence of autoimmune ACPA fine-specificities (here detected by the CCP2 test) with a well-known SE-association (26). Smoking on the other hand, a major risk factor for both RA (2, 25) and PD (43), associated significantly with anti-CPP3 antibodies even within the CCP2+ subset. In line with this observation, we have previously seen an increased prevalence of PD specifically in ACPA+ RA patients who smoke (44). Furthermore, anti-CPP3 IgG associated with higher baseline pain, which was not seen for anti-CCP2 IgG. We speculate that this association may reflect a pain-mediating effect of the anti-CPP3 antibodies, something that has previously been shown for ACPA in a mouse model (45).

Importantly, for the first time, we identified gingiva-residing B cells reactive to the citrullinated *Pg* CPP3 peptide. A number of CPP3+ and CPP3- gingival tissue B-cell clones also showed reactivity with citrullinated peptides derived from human proteins, in particular filaggrin and histone-4, known targets of the ACPA response (1, 46). Notably, the first ACPA test, the so-called anti-perinuclear factor, detected antibodies in RA sera that bound human buccal mucosa, later identified as citrullinated filaggrin (47). Moreover, NETosis with the release of citrullinated histones is a prominent feature of PD (41, 48), and presence of ACPA targeting citrullinated histones have been described in PD (49), in support of a link between periodontal infection and RA-related autoimmunity.

The GT mAbs frequently bound both citrullinated and corresponding arginine-containing peptides, in line with a previous report demonstrating elevated levels of antibodies recognizing both citrullinated and arginine-containing RA candidate autoantigens in PD patients *versus* non-PD controls (50). Regarding the high frequency of gingival tissue B cells with unspecific polyreactivity observed in our study, we speculate that this could reflect the bacteria-rich environment. Polyreactive germline-encoded IgM that bind both microbes and self-proteins are present in the natural antibody repertoire, and thought to have dual functions in immune defence and -homeostasis (51). Interestingly, the polyreactive gingival tissue clones were class-switched IgG or IgA with SHM, something that has previously also been described for polyreactive broadly neutralizing anti-HIV antibodies (52). Whether the polyreactive gingival tissue clones have functions in immune defence and homeostasis is not known and will be further investigated.

In addition to the CPP3+ gingival tissue B-cell clones, we identified an antibody derived from an RA blood memory B cell that showed cross-reactivity between *Pg* CPP3, human citrullinated peptides and the CCP2 peptide(s) used in the gold standard clinical test for RA. Peptide alignment pinpoints a shared “citrulline-motif”, previously described for this antibody (53), which could explain the cross-reactivity. In line with our data, Li et al. generated CCP2+ mAbs from circulating plasmablasts of ACPA+ RA patients, and could show cross-reactivity to *Pg* outer membrane proteins. Interestingly, the CCP2-reactivity was lost in the germline version of the antibody while reactivity to *Pg* remained (54), suggesting that autoimmunity may have evolved from an initial immune response against the bacteria. Future studies should address the clonal expansion of citrulline-reactive B cells in gingival tissue *versus* blood and joints.

**Figure 5:**
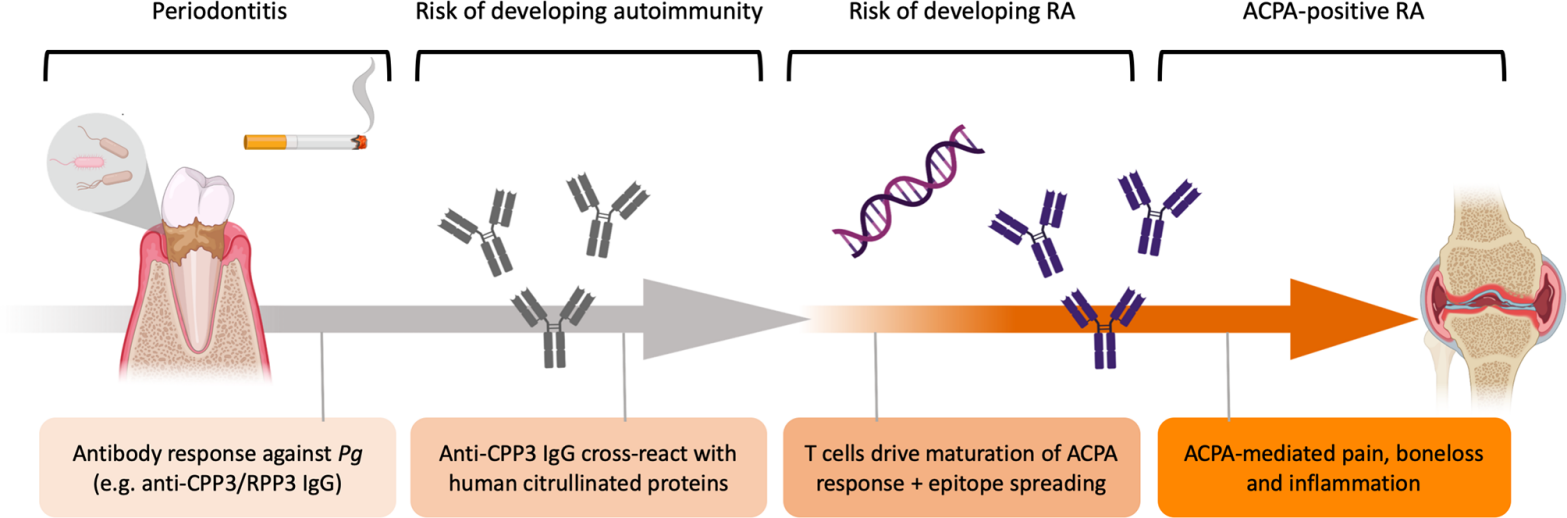
Schematic illustration of the etiological hypothesis linking *Pg* to ACPA+ RA. *P. gingivalis* drives gingival inflammation with increased protein citrullination, in the context of cigarette smoking. Immune responses against *Pg* includes anti-*P.*PAD antibody responses, such as anti-CPP3/RPP3 IgG, which could cross-react with human citrullinated proteins exposed in the inflamed periodontium by mechanisms of molecular mimicry. In genetically susceptible individuals (e.g. *HLA-DRB1* SE), autoreactive T cells drive affinity maturation of the ACPA response targeting self-proteins. High-affinity autoreactive ACPA subsequently bind proteins expressed in the joints and trigger pain, bone loss and synovial inflammation.

Based on our serological and molecular data, we propose an etiological model where *Pg* triggers loss of citrulline-tolerance, *via* production of anti-*Pg* antibodies (i.e. RPP3/CPP3) with a potential to cross-react with human citrullinated proteins by mechanisms of molecular mimicry. In genetically susceptible individuals (i.e. *HLA-DRB1* SE), autoreactive T cells may drive affinity maturation of this ACPA response towards citrullinated self-proteins (**Figure 5**).

An important technical achievement in our study, was the successful isolation of live B cells from frozen gingival biopsies. A similar protocol has been used for single-cell analyses of cells dissociated from cryopreserved synovial tissue (55). However, we observed lower leukocyte yields in our study, which may be explained by long-term storage at -80, and the enzymatic digestion may have degraded cell surface markers, hampering characterization of B-cell subsets from frozen biopsies. Still, B cells from one of the frozen gingival biopsies were successfully used to generate recombinant mAbs.

Analysis of B cells from fresh gingival tissue biopsies showed presence of both memory and plasma cells, in line with previous reports (56). Notably, our sorting strategy focused on CD19+ cells, yet recent reports have described long-lived CD19- plasma cells in bone marrow and intestine (57, 58). When analysing CD138 expression on CD19-/CD3-/CD14- cells in two of the GT biopsies, we were not able to detect a clear population (data not shown). However, patients were few and cell numbers low, thus further investigation is needed to examine the possible presence of long-lived CD19- plasma cells in inflamed gingiva. Our Ig repertoire analysis, demonstrating high numbers of SHM and replacement mutations in the CDRs, are supported by deep sequencing data of PD gingival tissue biopsies, revealing upregulation of genes involved in B-cell activation (59), and suggests antigen-dependent and T-cell driven B-cell responses in the inflamed periodontium. Although we observed some differences regarding B-cell subsets and BCR repertoire between ACPA+ RA/PD and non-RA/PD patients, we could not draw any conclusions due to the small cohort and inclusion of both frozen and fresh biopsies. In addition, we lacked detailed information on the patients donating gingival tissues, including pocked depth at the site of surgery, presence of *Pg*, age and smoking status, which would be relevant information if making such comparisons.

Another limitation of our study is the lack of periodontal data in the EIRA cohort, which prevented us from studying CPP3/RPP3 IgG in relation to PD status. Also, we did not detect any CCP2+ B cells in gingival tissue, but considering how rare CCP2+ B cells are (31, 60), and the limited number of GT mAbs analysed in our study (n=63 from two PD patients), the presence of CCP2+ B cells in the gum mucosa should be further explored.

Taken together, we show the presence of citrulline-reactive gingival tissue B cells, cross-reactivity between *P. gingivalis* and human citrullinated peptides on a monoclonal level, and an elevated antibody response to citrullinated *P.*PAD in CCP2+ RA patients, supporting a scenario where the ACPA response may be partly triggered by oral infection.

## Data Availability Statement

The raw data supporting the conclusions of this article will be made available by the authors, upon reasonable request.

## Ethics Statement

The studies involving human participants were reviewed and approved by Regional Ethics Review Board Stockholm, Stockholm, Sweden. The patients/participants provided their informed consent to participate in this study. In line with Swedish law, patient consent in the EIRA cohort was documented in the medical records by respective treating physician. This was done after the patient had received information about the study and after approving participation (consent) in the study.

## Author Contributions

NS, CV, and NK performed a majority of the laboratory work, with practical guidance from NSi, KA, and KL, and additional scientific guidance from CG and VM. XJ and BB performed association and correlation statistics. EK and LI contributed to ELISA screening and validation. MH and LM-A generated the multiplex array data. RS contributed to the mAb production. SS retrieved EIRA clinical data from the Swedish Rheumatology Quality Register. RH provided the Cit-C1 peptide and related validation. FS provided the BVCA1 Ig sequence, and supervised the work of LP. TY-L had overall responsibility for the collection of GT biopsies and supervised the work of KE, and together with AH, GJ, and AC identified PD and RA/PD patients for the study. JR analysed EIRA clinical data. LA administrates the EIRA study and supervised the work of XJ, BB, and SS. LK administrates the EIRA study together with LA, and initiated development of the multiplex array. LP identified and cloned BVCA1. KA, VM, and CG contributed to the design, discussions and interpretation of data in relation to the B cell, Ig and mAb experiments. KL conceived the study, supervised the work of NS, CV, NK, and EK, and had overall responsibility for the study, including writing the manuscript together with NS and CV. All authors have read, critically reviewed, and approved the final manuscript.

## Funding

This work was supported by grants from the Swedish Research Council (2017-01696), King Gustav V:s 80-year foundation (FAI-2016-0273), the Swedish Rheumatism foundation (R-931647), Professor Nanna Svartz’s foundation (2019-00292), and the EU/EFPIA Innovative Medicines Initiative Joint Undertaking RTCure (777357). FS and the Institute for Research in Biomedicine are supported by the Helmut Horten Foundation.

## Conflict of Interest

Karolinska Institutet has been a partner with Thermo Fisher Scientific within the Innovative Medicines Initiative BTCure, a public–private partnership between the EU and the European Federation of Pharmaceutical Industries (www.BTcure.eu). Thermo Fisher Scientific has contributed to this consortium with in-kind contributions for the development of the multiplex assay used in the study. LM-A is employed by Thermo Fischer Scientific. JR is member of the Thermo Fisher Scientific advisory board. KL is co-inventor of patent: US12/524,465, describing the diagnostic use of the CEP-1 epitope. RH is a co-inventor of patent: US Patent 7 148 020, protecting the use of the CitC1 peptide. GJ was employed by Praktikertjänst AB.

The remaining authors declare that the research was conducted in the absence of any commercial or financial relationships that could be construed as a potential conflict of interest.

## Publisher’s Note

All claims expressed in this article are solely those of the authors and do not necessarily represent those of their affiliated organizations, or those of the publisher, the editors and the reviewers. Any product that may be evaluated in this article, or claim that may be made by its manufacturer, is not guaranteed or endorsed by the publisher.

## Abbreviations

ACPA, anti-citrullinated protein antibodies; CCP, cyclic citrullinated peptide; CDR, complementary determining region; CPP3, citrullinated *Porphyromonas gingivalis *peptidylarginine deiminase peptide 3; EIRA, epidemiological investigation of rheumatoid arthritis; GT, gingival tissue; mAb, monoclonal antibody; *P.* PAD, *Porphyromonas gingivalis* peptidylarginine deiminase; PD, periodontitis; *Pg*, *Porphyromonas gingivalis*; RA, rheumatoid arthritis; RPP3, arginine (R)-containing *Porphyromonas gingivalis* peptide 3; SE, shared epitope (specific *HLA-DRB1* alleles); SHM, somatic hypermutation; VH, variable heavy (chain); VL, variable light (chain).
