# Supplementary material for: Antibodies to a Citrullinated Porphyromonas gingivalis Epitope Are Increased in Early Rheumatoid Arthritis, and Can Be Produced by Gingival Tissue B Cells: Implications for a Bacterial Origin in RA Etiology
Source: Front Immunol. 2022 Apr 20;13:804822. doi: 10.3389/fimmu.2022.804822 (PMC9066602; doi:10.3389/fimmu.2022.804822)
Supplement: Supplementary file 1 [file DataSheet_1.pdf]

SUPPLEMENTARY METHODS

Flow cytometry

Dissociated gingival tissue cells were re-suspended in 500µl PBS, 1% HS and labelled for flow cytometry. For viability, cells were stained with 7-aminoactinomycin D Viability Staining Solution (BioLegend) to detect dead/dying cells. Cells from GT01-GT06 were stained with: anti-CD3 Per-Cy 5.5 (clone UCHT1, BD), anti-CD14 Per-Cy 5.5 (Clone MϕP9, BD), anti-CD19 BV421 (Clone HIB19, BD), anti-CD138 APC (clone MI15, BD), and anti-CD27 APC-H7 (Clone M-T271, BD), and cells from GT07-GT10 were stained with: anti-CD3 APC-H7 (Clone SK1, BD), anti-CD14 APC-H7 (Clone MϕP9, BD), anti-CD19 BV421 (Clone HIB19, BD), anti-CD27 BV786 (Clone L128, BD), and anti-IgD FITC (Clone IA6-2, BD), for 30 min at 4°C. Cells were then washed (30ml PBS-HS), centrifuged (8 min, 400g, 4°C), re-suspended in 500µl PBS, 1% HS, and analyzed/sorted by flow cytometry (BD Influx), in accordance with guidelines described in Cossarizza et al (1). The lymphocyte gate was set based on the distribution of cells in SSC/FSC plots. Single viable CD3-CD14-CD19+ GT B cells were sorted (from all eight GT biopsies) into 96-well PCR plates, and stored at -80 until further processed. Memory B cells (CD19+CD27+CD138-) and plasma cells (CD19+CD27+CD138+) were analysed in two of the fresh biopsies (GT01 from a PD patient with ACPA+RA and GT03 from a PD patient without RA), while memory (CD19+CD27+IgD-), naïve (CD19+CD27-IgD+) and unswitched memory B cells (CD19+CD27+IgD+) were analysed in the two other fresh biopsies (GT10 from a PD patient with ACPA+RA and GT09 from a PD patient without RA).

Citrullinated peptide ELISAs

Monoclonal antibodies were analyzed for reactivity against synthetics citrullinated peptides (Innovagen AB, Lund, Sweden) derived from *Pg* PAD (CPP3) and human α-enolase (CEP-1), fibrinogen (Cit-$Fib_{36\text{-}50}$), vimentin (Cit-$Vim_{60\text{-}75}$), filaggrin (Cit-$Fil_{302\text{-}324}$) and histone 4 (Cit-$H4_{14\text{-}34}$ and Cit-$H4_{31\text{-}50}$), and the corresponding arginine-containing versions (see supplementary table 1 for peptide sequences). Briefly, high-binding 96-well plates (MaxiSorp, Nunc, Thermo Fisher Scientific, Roskilde, Denmark) were coated with the peptide diluted in carbonate buffer, pH 9.6 (CPP3 at 5µg/ml; CEP-1 at 2.5µg/ml) and incubated over night at 4°C, washed (PBS, 0.05% Tween) and blocked (1% BSA in PBS) for 1h at room temperature (RT). Alternatively, 96-wells Streptavidine plates (Pierce, Thermo Scientific, Rockford, USA) were washed (PBS, 0.05% Tween) and coated with biotinylated peptide (Cit-$Fib_{36\text{-}50}$ and Cit-$Vim_{60\text{-}75}$ at 1µg/ml; Cit-$Fil_{302\text{-}324}$ at 2µg/ml; and Cit-$H4_{14\text{-}34}$ and Cit-$H4_{31\text{-}50}$ at 2.5µg/ml) for 1h, RT. Monoclonal antibodies were added in single wells at 5µg/ml, diluted in RIA buffer (10mM Tris, 1% BSA, 350mM NaCl, 1% Triton-X, 0.5% sodium deoxycholate, 0.1% SDS), and incubated for 1h, RT. A standard curve (serial dilution of a positive serum pool) for each peptide ELISA, and a blank (RIA buffer), were included on all plates. Plates were washed (PBS, 0.05% Tween) and incubated with horse radish peroxidase (HRP)-conjugated goat anti-human IgG (Jacksson, Thermo Fisher Scientific, Stockholm, Sweden), diluted 1:10,000 in RIA buffer, for 1h at RT. After another wash (PBS, 0.05% Tween), TMB substrate (Sigma-Aldrich, St Louis, MO, USA) was added for approximately 15 minutes. The reaction was stopped by adding 0.5M H_2SO_4, and absorbance determined at 450nm with reference filter at 650nm. Positive mAbs (OD > 0.5) were re-analyzed in duplicates, 2-step dilution.

Polyreactivity ELISA

Polyreactivity was analyzed in an ELISA utilizing the soluble membrane protein (SMP) fraction from Hek293 cells (fractionated using ProteoExtract subcellular proteome extraction kit, Calbiochem, Darmstadt, Germany) as coating antigen. In brief, high-binding plates (MaxiSorp, Nunc) were coated with the Hek293 SMP fraction at 5μg/ml (diluted in carbonate buffer, pH 9.6), and incubated over night at 4°C, washed (PBS, 0.05% Tween) and blocked (PBS, 1% BSA) for 1h, RT. Monoclonal antibodies were added in single wells at 5μg/ml (diluted in PBS, 0.1% BSA) and incubated for 1h, RT. A previously identified polyreactive monoclonal antibody was added in serial dilution (starting at 20μg/ml, 1:2 dilution steps) as a standard, and a blank (PBS, 0.1% BSA) was included. Plates were washed (PBS, 0.05% Tween) and incubated with HRP-conjugated goat anti-human IgG (Jackson), diluted 1:10,000, for 1h at RT, washed again (PBS, 0.05% Tween), and TMB substrate (Sigma) was added for approximately 20 minutes. The reaction was stopped with 0.5M H_2SO_4, and absorbance measured at 450nm with reference filter at 650nm. Positive mAbs (OD > 0.5) were re-analyzed in duplicates, 2-step dilution.

Reference

[1] Cossarizza A, Chang HD, Radbruch A, Akdis M, Andra I, Annunziato F et al., Guidelines for use of flow cytometry and cell sorting in immunological studies (third edition). Eur J Immunol. 2021;51(12): 2708-3145. doi: 10.1002/eji.202170126
