# Supplementary material for: Antibodies to a Citrullinated Porphyromonas gingivalis Epitope Are Increased in Early Rheumatoid Arthritis, and Can Be Produced by Gingival Tissue B Cells: Implications for a Bacterial Origin in RA Etiology
Source: Front Immunol. 2022 Apr 20;13:804822. doi: 10.3389/fimmu.2022.804822 (PMC9066602; doi:10.3389/fimmu.2022.804822)
Supplement: Supplementary file 2 [file DataSheet_2.pdf]

Supplementary Table 1. Citrullinated peptides used on the multiplex peptide array and/or in ELISA

Peptide	Protein	Amino acid sequence	References
**CPP3**	*P.*PAD	C-AKTDSYWTCITDYTGWFAMYD-C	(1)
Cit-C1	collagen type II	(GPO)5-GLOGACITGLTGCITOGDAGPQGKVGPS-$(GPO)_2$-GKKYG	(2)
**CEP-1**	α-enolase	C-KIHACITEIFDSCITGNPTVE-C	(3)
Cit-fibα$_{563-583}$	fibrinogen α	HHPGIAEFPSCITGKSSSYSKQF	(4, 5)
Cit-fibα$_{580-600}$	fibrinogen α	SKQFTSSTSYNCITGDSTFESKS	(5, 6)
**Cit-fibß$_{36-52}$**	fibrinogen ß	NEEGFFSACITGHRPLDKK	(7)
Cit-Vim$_{2-17}$	vimentin	STCITSVSSSSYCITCITMFGG	(8)
**Cit-Vim$_{60-75}$**	vimentin	VYATCITSSAVCITLCITSSVP	(7)
**Cfc1-cyc**	filaggrin	HQCHQESTCITGRSRGRCGRSGS	(9)
**Cit-H4$_{14-34}$**	histone H4	$(GAKCITHCITKVLCITDNIQGITKPAI)_4K_2K\beta A$*	(10)
**Cit-H4$_{31-50}$**	histone H4	$(KPAICITCITLACITCITGGVKCITISGLI)_4K_2K\beta A$*	(10)

All peptides, with the exception of the two Cit-H4 peptides, were included on the multiplex microarray; peptides in bold were used in ELISA. CPP3 = citrullinated *P.*PAD peptide 3; Cit-C1 = citrullinated triple helical C1 epitope on collagen type II; CEP-1 = citrullinated α-enolase peptide 1; Cfc1-cyc (cyclic citrullinated filaggrin peptide 1); Cit/CIT = citrullinated/citrulline; *P.*PAD = *Porphyromonas gingivalis* peptidyl arginine deiminase. *Multiple antigen peptides bearing four identical sequences on a lysine scaffold.

References (supplementary table 1)

[1] Quirke AM, Lugli EB, Wegner N, Hamilton BC, Charles P, Chowdhury M, et al., Heightened immune response to autocitrullinated Porphyromonas gingivalis peptidylarginine deiminase: a potential mechanism for breaching immunologic tolerance in rheumatoid arthritis. Ann Rheum Dis. 2014;73(1): 263-9. doi:10.1136/annrheumdis-2012-202726

[2] Burkhardt H, Sehnert B, Bockermann R, Engström A, Kalden JR, and Holmdahl R, Humoral immune response to citrullinated collagen type II determinants in early rheumatoid arthritis. Eur J Immunol. 2005;35(5): 1643-52. doi:10.1002/eji.200526000

[3] Lundberg K, Kinloch A, Fisher BA, Wegner N, Wait R, Charles P, et al., Antibodies to citrullinated alpha-enolase peptide 1 are specific for rheumatoid arthritis and cross-react with bacterial enolase. Arthritis Rheum. 2008;58(10): 3009-19. doi:10.1002/art.23936

[4] Hermansson M, Artemenko K, Ossipova E, Eriksson H, Lengqvist J, Makrygiannakis D, et al., MS analysis of rheumatoid arthritic synovial tissue identifies specific citrullination sites on fibrinogen. Proteomics Clin Appl. 2010;4(5): 511-8. doi:10.1002/prca.200900088

[5] Too CL, Murad S, Hansson M, Alm LM, Dhaliwal JS, Holmdahl R, et al., Differences in the Spectrum of Anti-Citrullinated Protein Antibody Fine Specificities Between Malaysian and Swedish Patients With Rheumatoid Arthritis: Implications for Disease Pathogenesis. Arthritis Rheumatol. 2017;69(1): 58-69. doi:10.1002/art.39827
[6] Hansson M, Mathsson L, Schlederer T, Israelsson L, Matsson P, Nogueira L, et al., Validation of a multiplex chip-based assay for the detection of autoantibodies against citrullinated peptides. Arthritis Res Ther. 2012;14(5): R201. doi:10.1186/ar4039
[7] Verpoort KN, Cheung K, Ioan-Facsinay A, van der Helm-van Mil AH, de Vries-Bouwstra JK, Allaart CF, et al., Fine specificity of the anti-citrullinated protein antibody response is influenced by the shared epitope alleles. Arthritis Rheum. 2007;56(12): 3949-52. doi:10.1002/art.23127
[8] Snir O, Widhe M, Hermansson M, von Spee C, Lindberg J, Hensen S, et al., Antibodies to several citrullinated antigens are enriched in the joints of rheumatoid arthritis patients. Arthritis Rheum. 2010;62(1): 44-52. doi:10.1002/art.25036
[9] Schellekens GA, Visser H, de Jong BA, van den Hoogen FH, Hazes JM, Breedveld FC, et al., The diagnostic properties of rheumatoid arthritis antibodies recognizing a cyclic citrullinated peptide. Arthritis Rheum. 2000;43(1): 155-63. doi:10.1002/1529-0131(200001)43:1<155::Aid-anr20>3.0.Co;2-3
[10] Pratesi F, Dioni I, Tommasi C, Alcaro MC, Paolini I, Barbetti F, et al., Antibodies from patients with rheumatoid arthritis target citrullinated histone 4 contained in neutrophils extracellular traps. Ann Rheum Dis. 2014;73(7): 1414-22. doi:10.1136/annrheumdis-2012-202765

Supplementary table 2. Patient-reported baseline pain and global assessment, in EIRA RA patients stratified for anti-CPP3 and anti-CCP2 antibody status

**Baseline Pain** (median VAS)		
**CPP3+/CCP2+** (N=156)	**CPP3-/CCP2+** (N=831)	**P-values**
60	50	**0.018**<br>**0.023***<br>**0.031****
**Baseline global assessment** (median VAS)		
**CPP3+/CCP2+** (N=156)	**CPP3-/CCP2+** (N=834)	**P-values**
55.5	51	**0.042**<br>**0.047***<br>0.052**

Median values for patient-reported pain and global assessment (using visual analogue scale: 0-100) at baseline, in CPP3+/CCP2+ *versus* CPP3-/CCP2+ RA (CPP3 IgG status based on the 98th percentile cut-off). Significant p-values are shown in bold. *p-values adjusted for smoking status, **p-values adjusted for age, sex and smoking status (multivariate analyses). N = number of patients with clinical data available.

Supplementary Table 3. Differences in patient-reported pain and global assessment, during five-years follow-up of EIRA RA patients, stratified for CPP3 or CCP2 antibody status

	**Time point**	**N**	**CPP3+**	**CPP3-**	**P-value**	**CCP2+**	**CCP2-**	**P-value**
Pain*	Baseline	1600	60	50	**0.009**	51	51	0.619
	3 months	1329	29.5	27	0.14	28	26	0.597
	6 months	1195	25.5	25	0.662	26	24	0.921
	12 months	1449	22	22	0.741	22	21	0.146
	24 months	1357	19	22	0.126	22	21	0.872
	36 months	885	19.5	25	0.243	27	21	**0.028**
	48 months	652	28.5	24	0.243	25	21.5	0.107
	60 months	905	22	24.5	0.623	25	22.5	0.092
Patient global assessment**	Baseline	1553	56	51	**0.036**	51	51	0.957
	3 months	1328	27	29	0.948	29	29	0.711
	6 months	1199	27	26	0.598	26	26	0.893
	12 months	1452	23	23	0.862	23	21	0.19
	24 months	1363	20	22	0.298	22	21	0.6
	36 months	892	21	26	0.221	27.5	23	**0.026**
	48 months	653	31.5	24	0.111	26	23	0.285
	60 months	908	26	22	0.603	25	18	0.104

Median values for patient-reported *pain and **patient global assessment (using visual analogue scale: 0-100) at baseline and follow-up, in CPP3+ *versus* CPP3- RA (CPP3 IgG status based on the 98th percentile cut-off), and in CCP2+ *versus* CCP2- RA. Significant p-values are shown in bold. N = number of patients with clinical data available.

Supplementary Table 4. Differences in ESR and CRP, during five-years follow-up of EIRA RA patients, stratified for anti-CPP3 or anti-CCP2 antibody status

	**Time point**	**N**	**CPP3+**	**CPP3-**	**P-value**	**CCP2+**	**CCP2-**	**P-value**
ESR*	Baseline	1528	32	27	0.0721	29	24	**<0.0001**
	3 months	1282	16	14	0.0812	16	12	**<0.0001**
	6 months	1158	13	12	0.3500	14	10	**<0.0001**
	12 months	1393	15	12	**0.0180**	13	10	**<0.0001**
	24 months	1324	13	12	0.3428	12	11	**0.0059**
	36 months	869	12	12	0.5132	13	10	**0.0005**
	48 months	623	14	13	**0.0493**	14	12	**0.0192**
	60 months	882	13	13	0.8428	14.5	11	**<0.0001**
CRP**	Baseline	1565	18	14	0.1892	15	13	0.0753
	3 months	1329	8	8	0.7191	8	8	**0.0121**
	6 months	1192	8	7	0.3700	8	7	0.1319
	12 months	1437	8	7	0.2028	7	7	0.0702
	24 months	1345	7	7	0.8296	7	7	0.5780
	36 months	873	5	5	0.8571	6	4	**0.0044**
	48 months	648	4	4	0.1981	5	4	**0.0139**
	60 months	867	5	5	0.6741	5	4	**0.0114**

Median values for *erythrocyte sedimentation rate (ESR) (mm/hour) and **C-reactive protein (CRP) levels (mg/L) are shown at baseline and follow-up, in CPP3+ *versus* CPP3- RA (CPP3 IgG status based on the 98th percentile cut-off), and in CCP2+ *versus* CCP2- RA. Significant p-values are shown in bold. N = number of patients with clinical data available.

Supplementary Table 5. Differences in swollen- and tender joint count, during five-years follow-up of EIRA RA patients, stratified for anti-CPP3 or anti-CCP2 antibody status

	**Time point**	**N**	**CPP3+**	**CPP3-**	**P-value**	**CCP2+**	**CCP2-**	**P-value**
swollen joint count*	Baseline	1584	10	9	0.6550	8	10	**<0.0001**
	3 months	1350	3	2	0.1378	2	2	0.0916
	6 months	1206	1	1	0.6295	2	1	**0.0269**
	12 months	1470	1	1	0.1159	1	0	**<0.0001**
	24 months	1386	0	0	0.6167	0	0	**0.0010**
	36 months	916	0	1	0.8835	1	0	**<0.0001**
	48 months	673	1	0	0.0980	1	0	**0.0088**
	60 months	929	0	0	0.8175	1	0	**<0.0001**
tender joint count**	Baseline	1584	8	8	0.9217	7	8	**0.0003**
	3 months	1350	2	2	0.3016	2	2	0.1963
	6 months	1206	2	1	0.6481	1	1	0.5451
	12 months	1469	1	1	0.5506	1	1	0.3413
	24 months	1384	1	0	0.8000	1	1	0.5934
	36 months	918	0	1	0.1041	1	0	0.2642
	48 months	674	1	1	0.3263	1	0	0.1307
	60 months	930	0	1	0.2665	1	0	**0.0400**

Median values for *tender- and **swollen joint counts (for 28 joints) are shown at baseline and follow-up, in CPP3-positive *versus* CPP3-negative RA (CPP3 IgG status based on the 98th percentile cut-off), and in CCP2-positive *versus* CCP2-negative RA. Significant p-values are shown in bold. N = number of patients with clinical data available.

Supplementary Table 6. Differences in DAS28 and HAQ, during five-years follow-up of EIRA RA patients, stratified for anti-CPP3 or anti-CCP2 antibody status

	**Time point**	**N**	**CPP3+**	**CPP3-**	**P-value**	**CCP2+**	**CCP2-**	**P-value**
DAS28*	Baseline	1482	5.52	5.32	0.1848	5.31	5.39	0.2410
	3 months	1261	3.65	3.49	0.6609	3.56	3.41	0.0520
	6 months	1136	3.17	3.11	0.6656	3.16	3.05	0.1962
	12 months	1363	3.02	2.78	0.0841	2.87	2.68	**0.0016**
	24 months	1289	2.86	2.67	0.6124	2.73	2.63	0.1382
	36 months	831	2.67	2.87	0.1698	2.92	2.68	**0.0041**
	48 months	582	3.11	2.85	0.1131	2.93	2.69	**0.0484**
	60 months	851	2.5	2.73	0.6138	2.88	2.38	**<0.0001**
HAQ**	Baseline	1527	1.0	1.0	0.2329	1.0	1.0	0.9515
	3 months	1297	0.5	0.5	0.6855	0.5	0.5	0.6956
	6 months	1171	0.5	0.5	0.8585	0.5	0.5	0.4013
	12 months	1416	0.38	0.38	0.6514	0.3	0.3	0.5084
	24 months	1331	0.38	0.38	0.3330	0.3	0.3	0.8632
	36 months	861	0.38	0.5	0.7326	0.5	0.3	0.6535
	48 months	636	0.63	0.5	0.4331	0.5	0.5	0.8422
	60 months	888	0.5	0.5	0.5593	0.5	0.3	0.5423

Median values for *disease activity score for 28 joints (DAS28) and **health assessment questionnaire (HAQ) functional impairment index are shown at baseline and follow-up, in CPP3+ *versus* CPP3- RA (CPP3 IgG status based on the 98th percentile cut-off), and in CCP2+ *versus* CCP2- RA. Significant p-values are shown in bold. N = number of patients with clinical data available.

Supplementary Table 7. Non-GT monoclonal antibodies screened for CPP3-reactivity

mAb ID	Origin	Cell type	CCP2	Reference
1003:01C27	SF	mB	neg	
1003:01D42	SF	mB	neg	
1003:01G05	SF	mB	neg	
1003:02D03	SF	mB	neg	
1003:10A01	SF	mB	**pos**	(1)
1103:01A03	SF	mB	neg	
1103:01A04	SF	mB	neg	
1103:01B01	SF	mB	neg	
1103:01B02	SF	mB	neg	
1103:01B04	SF	mB	neg	
1103:01B08	SF	mB	neg	
1103:01E01	SF	mB	neg	
1103:01E02	SF	mB	neg	
1103:01E06	SF	mB	neg	
1103:01E08	SF	mB	neg	
1103:01F05	SF	mB	neg	
1103:01F08	SF	mB	neg	
1103:01F09	SF	mB	neg	
1103:01G05	SF	mB	neg	
1103:01H05	SF	mB	neg	
1103:01H11	SF	mB	neg	
1124:02B18	SF	mB	neg	
1276:01A09	SF	mB	neg	(2)
1276:01B07	SF	mB	neg	(2)
1276:01C09	SF	mB	neg	(2)
1276:01C11	SF	mB	neg	(2)
1276:01D10	SF	PC	neg	(2)
1276:01E06	SF	mB	neg	(2)
1276:01E09	SF	mB	neg	(2)
1276:01E11	SF	mB	neg	(2)
1276:01F04	SF	mB	neg	(2)
1276:01F10	SF	mB	neg	(2)
1276:01F11	SF	mB	neg	(2)
1276:01G08	SF	mB	neg	(2)
1276:01G09	SF	mB	neg	(2)
1276:01H09	SF	mB	neg	(2)
1276:01H10	SF	mB	neg	(2)
1276:02A05	SF	PC	neg	(2)
1276:02B10	SF	PC	neg	(2)
1276:02F04	SF	PC	neg	(2)

1276:02H01	SF	PC	neg	(2)
1276:02H02	SF	PC	neg	(2)
1276:05A01	SF	PC	neg	(2)
1276:05A02	SF	PC	neg	(2)
1276:05B01	SF	PC	neg	(2)
1276:05E05	SF	PC	neg	(2)
1276:05F06	SF	PC	neg	(2)
1276:05F10	SF	PC	neg	(2)
1276:05G05	SF	PC	neg	(2)
1276:05H05	SF	PC	neg	(2)
1276:06D06	SF	PC	neg	(2)
1276:08B02	SF	PC	neg	(2)
1276:08B06	SF	PC	neg	(2)
1276:08B10	SF	PC	neg	(2)
1276:08B11	SF	PC	neg	(2)
1276:08C09	SF	PC	neg	(2)
1276:08C11	SF	PC	neg	(2)
1276:08C12	SF	PC	neg	(2)
1276:08D01	SF	PC	neg	(2)
1276:08D11	SF	PC	neg	(2)
1276:08E10	SF	PC	neg	(2)
1276:08F06	SF	PC	neg	(2)
1276:08F10	SF	PC	neg	(2)
1276:08F11	SF	PC	neg	(2)
1276:08H04	SF	PC	neg	(2)
1276:08H06	SF	PC	neg	(2)
1276:08H06	SF	PC	neg	(2)
1276:08H07	SF	PC	neg	(2)
1276:08H09	SF	PC	neg	(2)
1276:09A01	SF	PC	neg	(2)
1276:09D05	SF	PC	neg	(2)
1325:01B01	SF	mB	neg	(3)
1325:01B05	SF	mB	neg	(3)
1325:01B07	SF	mB	neg	(3)
1325:01B09	SF	PC	**pos**	(3)
1325:01C04	SF	mB	neg	(3)
1325:01C05	SF	mB	neg	(3)
1325:01F07	SF	mB	neg	(3)
1325:01H07	SF	mB	neg	(3)
1325:04C03	SF	PC	**pos**	(3)
1325:05C06	SF	PC	**pos**	(3)
1325:07E07	SF	PC	**pos**	(3)
1362:01E02	SF	mB	neg	(2)

1362:03A03	SF	PC	neg	(2)
1362:03A04	SF	PC	neg	(2)
1362:03A08	SF	PC	neg	(2)
1362:03B02	SF	PC	neg	(2)
1362:03B04	SF	PC	neg	(2)
1362:03B05	SF	PC	neg	(2)
1362:03D08	SF	mB	neg	(2)
1362:03E06	SF	mB	neg	(2)
1362:03F04	SF	PC	neg	(2)
1362:03F12	SF	PC	neg	(2)
1362:03H05	SF	PC	neg	(2)
1362:03H07	SF	PC	neg	(2)
1362:06B09	SF	PC	neg	(2)
1362:06C03	SF	PC	neg	(2)
1362:06C04	SF	PC	neg	(2)
1362:06C06	SF	PC	neg	(2)
1362:06C07	SF	PC	neg	(2)
1362:06C08	SF	PC	neg	(2)
1362:06C09	SF	PC	neg	(2)
1362:06C10	SF	PC	neg	(2)
1362:07A05	SF	PC	neg	(2)
1362:07A07	SF	PC	neg	(2)
1362:07A09	SF	PC	neg	(2)
1362:07A10	SF	PC	neg	(2)
1362:07B01	SF	PC	neg	(2)
1362:07B02	SF	PC	neg	(2)
1362:07D09	SF	PC	neg	(2)
1362:07D10	SF	PC	neg	(2)
1362:07E03	SF	PC	neg	(2)
1362:07E04	SF	PC	neg	(2)
1362:07E06	SF	PC	neg	(2)
1362:07E07	SF	PC	neg	(2)
1362:07F10	SF	PC	neg	(2)
1362:07F11	SF	PC	neg	(2)
1362:07F12	SF	PC	neg	(2)
1362:07G06	SF	PC	neg	(2)
1362:07G08	SF	PC	neg	(2)
1362:07G12	SF	PC	neg	(2)
136207H03	SF	PC	neg	(2)
136207H06	SF	PC	neg	(2)
1444:01A09	SF	PC	neg	(2)
1444:01C04	SF	PC	neg	(2)
1444:01E07	SF	mB	neg	(2)

1444:01E09	SF	PC	neg	(2)
1444:01H04	SF	PC	neg	(2)
1444:02C08	SF	mB	neg	(2)
1444:02F07	SF	mB	neg	(2)
1444:02G09	SF	mB	neg	(2)
1444:03B06	SF	mB	neg	(2)
1444:04A05	SF	mB	neg	(2)
1444:04B08	SF	mB	neg	(2)
1444:04F02	SF	PC	neg	(2)
1444:04F10	SF	PC	neg	(2)
1444:10A07	SF	PC	neg	(2)
1444:10B08	SF	PC	neg	(2)
1444:10D11	SF	PC	neg	(2)
14sm:01B11	PB	CFC+ mB	neg	(4)
14CFCT2D09	PB	CFC+ mB	**pos**	(4)
14CFCT2H12	PB	CFC+ mB	**pos**	(4)
14CFCT3G09	PB	CFC+ mB	**pos**	(4)
37sm:01B12	PB	sw-mem	neg	(4)
37sm:01G08	PB	sw-mem	neg	(4)
37sm:01G12	PB	sw-mem	neg	(4)
37sm:01H02	PB	sw-mem	**pos**	(4)
37CEPT1A03	PB	CEP+ mB	neg	(4)
37CEPT1A07	PB	CEP+ mB	neg	(4)
37CEPT1E10	PB	CEP+ mB	neg	(4)
37CEPT1E11	PB	CEP+ mB	neg	(4)
37CEPT1F09	PB	CEP+ mB	neg	(4)
37CEPT1G09	PB	CEP+ mB	**pos**	(4)
37CEPT1H08	PB	CEP+ mB	neg	(4)
37CEPT1H09	PB	CEP+ mB	neg	(4)
37CEPT2B06	PB	CEP+ mB	neg	(4)
37CEPT2C04	PB	CEP+ mB	**pos**	(4)
37CEPT2E09	PB	CEP+ mB	neg	(4)
37CEPT2H03	PB	CEP+ mB	neg	(4)
37CEPT2H12	PB	CEP+ mB	neg	(4)
62sm:01A05	PB	sw-mem	neg	(4)
62sm:01B07	PB	sw-mem	neg	(4)
62sm:01B08	PB	sw-mem	neg	(4)
62sm:01B09	PB	sw-mem	neg	(4)
62sm:01C03	PB	sw-mem	neg	(4)
62sm:01C04	PB	sw-mem	neg	(4)
62sm:01D02	PB	sw-mem	neg	(4)
62sm:01E07	PB	sw-mem	neg	(4)
62sm:01F02	PB	sw-mem	neg	(4)

62sm:01F05	PB	sw-mem	neg	(4)
62sm:01H06	PB	sw-mem	neg	(4)
62sm:01H07	PB	sw-mem	neg	(4)
62CFCT1E04	PB	CFC+ mB	**pos**	(4)
62CFCT1H06	PB	CFC+ mB	neg	(4)
BVCA:01A01	PB	mB	**pos**	(2)

SF = synovial fluid; PB = peripheral blood; mB = memory B cell; PC = plasma cell; sw-mem = switched memory B cell; CFC+ = sorted with Cfc1-cyc tetramer staining (Cfc1-cyc = cyclic citrullinated filaggrin peptide); CEP+ = sorted with CEP-1 tetramer staining (CEP-1 = cyclic citrullinated α-enolase peptide 1); pos = positive in the CCP2 ELISA; neg = negative in the CCP2 ELISA.

References (supplementary table 7)

[1] Germar K, Fehres CM, Scherer HU, van Uden N, Pollastro S, Yeremenko N, et al., Generation and Characterization of Anti-Citrullinated Protein Antibody-Producing B Cell Clones From Rheumatoid Arthritis Patients. Arthritis Rheumatol. 2019;71(3): 340-350. doi:10.1002/art.40739

[2] Lloyd KA, Steen J, Amara K, Titcombe PJ, Israelsson L, Lundström SL, et al., Variable domain N-linked glycosylation and negative surface charge are key features of monoclonal ACPA: Implications for B-cell selection. Eur J Immunol. 2018;48(6): 1030-1045. doi:10.1002/eji.201747446

[3] Steen J, Forsström B, Sahlström P, Odowd V, Israelsson L, Krishnamurthy A, et al., Recognition of Amino Acid Motifs, Rather Than Specific Proteins, by Human Plasma Cell–Derived Monoclonal Antibodies to Posttranslationally Modified Proteins in Rheumatoid Arthritis. Arthritis Rheumatol. 2019;71(2): 196-209. doi:https://doi.org/10.1002/art.40699

[4] Titcombe PJ, Wigerblad G, Sippl N, Zhang N, Shmagel AK, Sahlström P, et al., Pathogenic Citrulline-Multispecific B Cell Receptor Clades in Rheumatoid Arthritis. Arthritis Rheumatol. 2018;70(12): 1933-1945. doi:10.1002/art.40590
