# Supplementary material for: Antibodies to a Citrullinated Porphyromonas gingivalis Epitope Are Increased in Early Rheumatoid Arthritis, and Can Be Produced by Gingival Tissue B Cells: Implications for a Bacterial Origin in RA Etiology
Source: Front Immunol. 2022 Apr 20;13:804822. doi: 10.3389/fimmu.2022.804822 (PMC9066602; doi:10.3389/fimmu.2022.804822)
Supplement: Supplementary file 3 [file DataSheet_3.pdf]

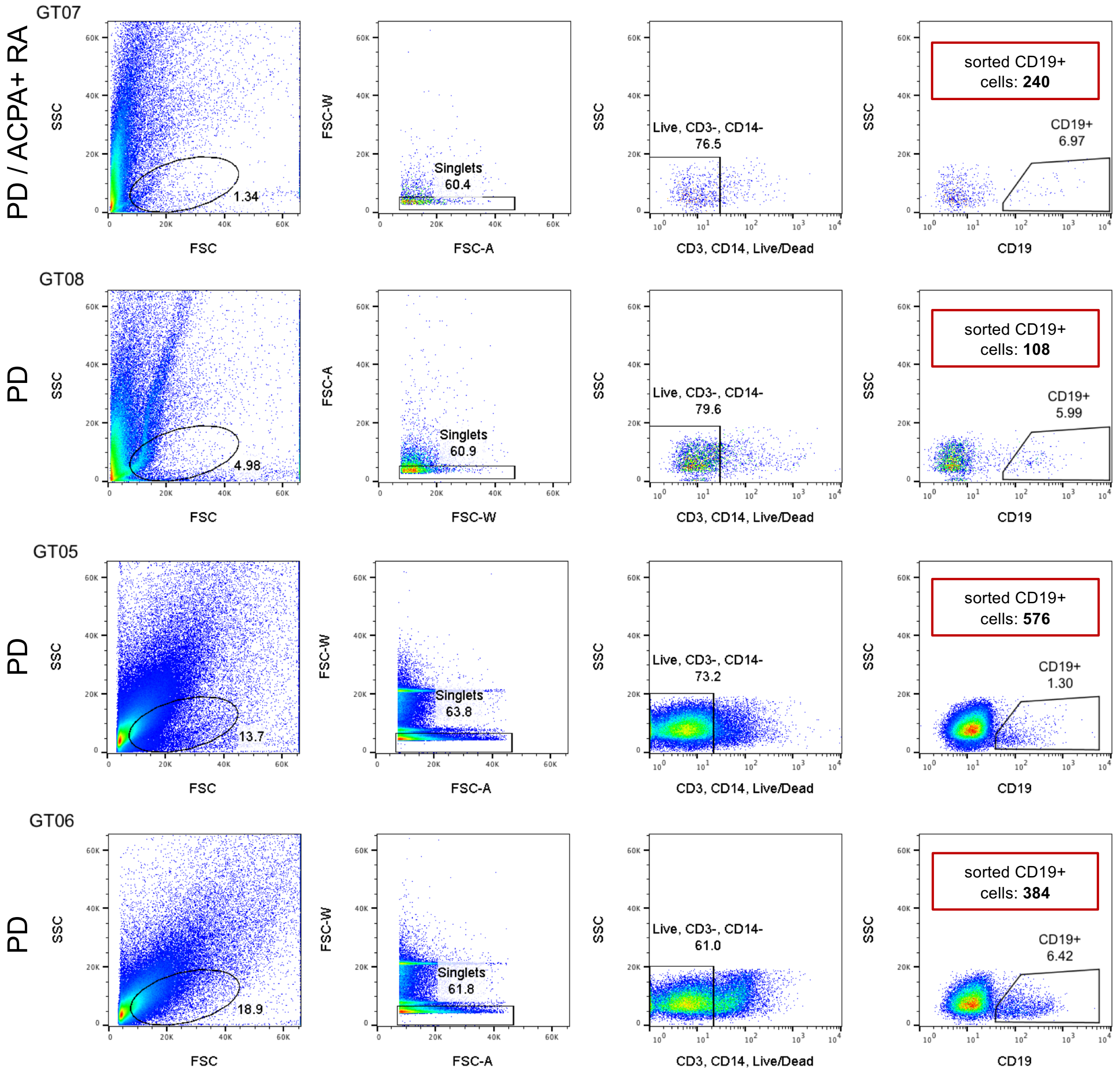

Supplementary figure 1. Flow cytometry sorting of CD19+ B cells from gingival tissue biopsies. Gating strategy and sorting results for CD19+ B cells from frozen GT biopsies of one PD patient with ACPA+ RA (GT07), and two PD patients without RA (GT08, GT05 and GT06); GT05 and GT06 were biopsies from the same PD patient. Red boxes show the number of CD19+ B cells sorted from each biopsy. FSC = forward scatter; SSC = side scatter; GT = gingival tissue; PD = periodontitis; ACPA = anti-citrullinated protein antibody; RA = rheumatoid arthritis.

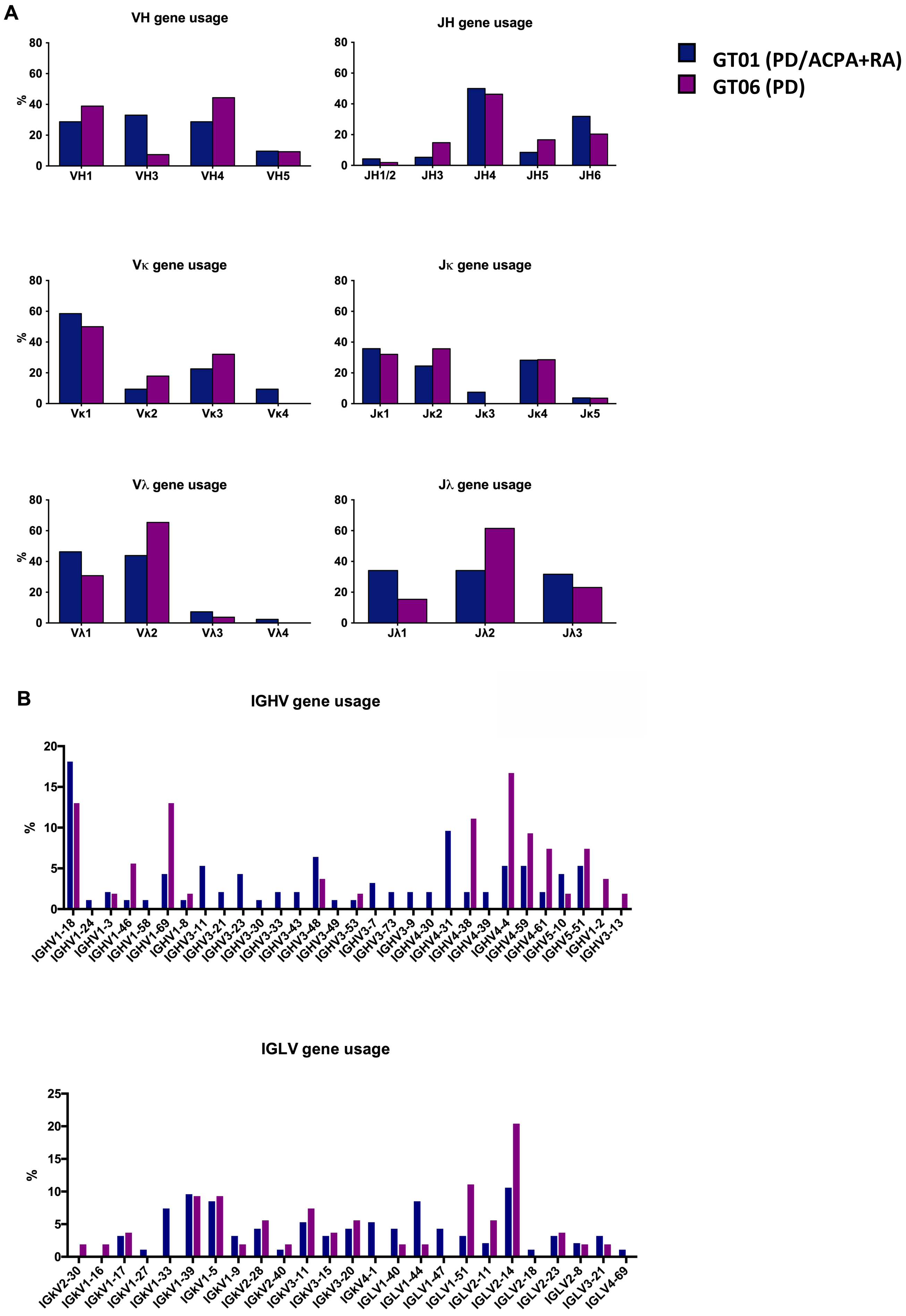
A
VH gene usage
JH gene usage
GT01 (PD/ACPA+RA)
GT06 (PD)
%
VH1
VH3
VH4
VH5
JH1/2
JH3
JH4
JH5
JH6
Vκ gene usage
Jκ gene usage
Vκ1
Vκ2
Vκ3
Vκ4
Jκ1
Jκ2
Jκ3
Jκ4
Jκ5
Vλ gene usage
Jλ gene usage
Vλ1
Vλ2
Vλ3
Vλ4
Jλ1
Jλ2
Jλ3
B
IGHV gene usage
IGHV1-18
IGHV1-24
IGHV1-3
IGHV1-46
IGHV1-58
IGHV1-69
IGHV1-8
IGHV3-11
IGHV3-21
IGHV3-23
IGHV3-30
IGHV3-33
IGHV3-43
IGHV3-48
IGHV3-49
IGHV3-53
IGHV3-7
IGHV3-73
IGHV3-9
IGHV4-30
IGHV4-31
IGHV4-38
IGHV4-39
IGHV4-4
IGHV4-59
IGHV4-61
IGHV5-10
IGHV5-51
IGHV1-2
IGHV3-13
IGLV gene usage
IGKV2-30
IGKV1-16
IGKV1-17
IGKV1-27
IGKV1-33
IGKV1-39
IGKV1-5
IGKV1-9
IGKV2-28
IGKV2-40
IGKV3-11
IGKV3-15
IGKV3-20
IGKV4-1
IGLV1-40
IGLV1-44
IGLV1-47
IGLV1-51
IGLV2-11
IGLV2-14
IGLV2-18
IGLV2-23
IGLV2-8
IGLV3-21
IGLV4-69

Supplementary figure 2. Immunoglobulin gene usage in gingival B cells from two PD patients. (**A**) Summary of paired Ig VH and VL (Vκ and Vλ), as well as JH and JL (Jκ and Jλ), family gene usage in single-cell sorted CD19+ B cells from a PD patient with ACPA+RA (GT01; n=94 B cells) and a PD patient without RA (GT06; n=54 B cells). (**B**) Ig VH and VL chain gene segment usage in GT01 and GT06; analysed by IgBLAST and IMGT/V-QUEST. V = variable gene segment; J = joining gene segment; H = heavy chain; L = light chain; Ig = immunoglobulin; ACPA = anti-citrullinated protein antibodies; GT = gingival tissue; PD = periodontitis; RA = rheumatoid arthritis.

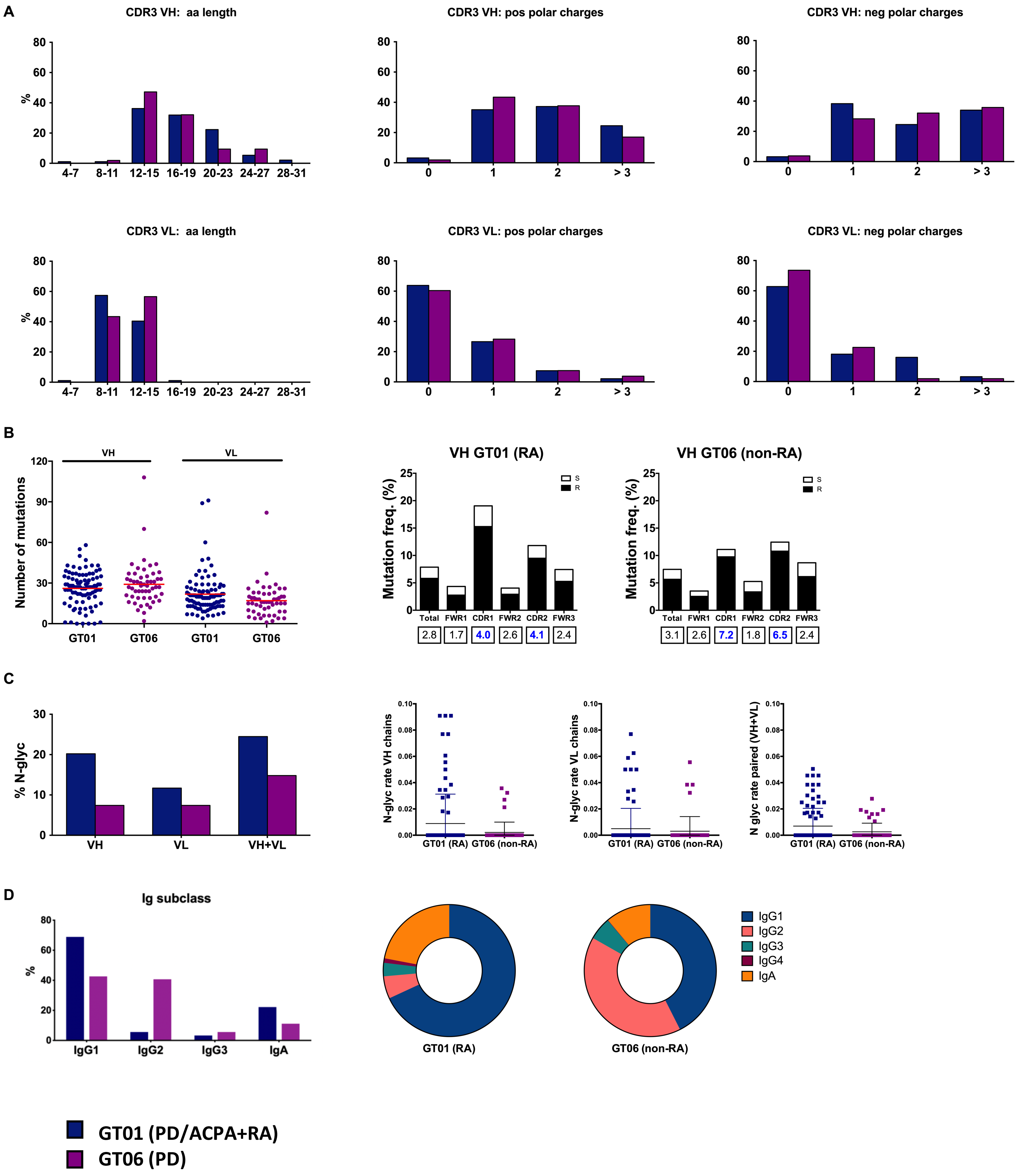
A
CDR3 VH: aa length
CDR3 VH: pos polar charges
CDR3 VH: neg polar charges
CDR3 VL: aa length
CDR3 VL: pos polar charges
CDR3 VL: neg polar charges
%
4-7 8-11 12-15 16-19 20-23 24-27 28-31
0 1 2 > 3
B
VH
VL
Number of mutations
GT01 GT06 GT01 GT06
VH GT01 (RA)
VH GT06 (non-RA)
Mutation freq. (%)
S
R
Total FWR1 CDR1 FWR2 CDR2 FWR3
2.8 1.7 4.0 2.6 4.1 2.4
3.1 2.6 7.2 1.8 6.5 2.4
C
% N-glyc
VH VL VH+VL
N-glyc rate VH chains
N-glyc rate VL chains
N glyc rate paired (VH+VL)
GT01 (RA) GT06 (non-RA)
D
Ig subclass
IgG1 IgG2 IgG3 IgA
GT01 (RA)
GT06 (non-RA)
IgG1
IgG2
IgG3
IgG4
IgA
GT01 (PD/ACPA+RA)
GT06 (PD)

Supplementary figure 3. Immunoglobulin variable gene characteristics and subclass distribution, in gingival B cells from two PD patients. (**A**) Overview of IgH (top panels) and IgL (bottom panels) CDR3 amino acid characteristics of CD19+ GT B cells from a PD patient with ACPA+RA (GT01; n=94 B cells) and a PD patient without RA (GT06; n=54 B cells); CDR3 length and positively/negatively charged amino acids (aa) are shown. (**B**) Total number of mutations in Ig VH and VL chains from GT01 and GT06 (left); frequency (%) of replacement (R) and silent (S) mutations, and R/S ratio, within VH and VL chains in FWRs and CDRs (right). (**C**) Frequency (%) of all sequences (irrespective of the number of SHM) containing N-glyc motifs (left), and consensus N-glyc motifs rates (right), for VH, VL, and paired sequences; glycosylation rates are depicted as the percentage of sequences containing N-glyc motifs adjusted for SHMs. (**D**) Ig subclass distribution in GT01 and GT06. CDR = complementary determining region; VH = variable heavy gene segment; VL = variable light gene segment; FWR = framework region; N-glyc = predicted N-linked glycosylation motifs (N-X-S/T); Ig = immunoglobulin; ACPA = anti-citrullinated protein antibodies; GT = gingival tissue; PD = periodontitis; RA = rheumatoid arthritis.
